# Effects of Online Teaching during the COVID-19 Pandemic on Nursing Students’ Intention to Join the Nursing Workforce: A Cross-Sectional Study

**DOI:** 10.3390/healthcare10081461

**Published:** 2022-08-03

**Authors:** Pei-Ti Hsu, Ya-Fang Ho

**Affiliations:** 1 Department of Nursing, Ching Kuo Institute of Management and Health, Keelung 20301, Taiwan; bettyhsu1@gmail.com; 2 School of Nursing, China Medical University, Taichung 406040, Taiwan; 3 Nursing Department, China Medical University Hospital, Taichung 404332, Taiwan

**Keywords:** online teaching, nursing student, COVID-19 pandemic, nursing work intention

## Abstract

Nursing education programs were interrupted and largely moved online as a result of the COVID-19 pandemic. The purpose of this study was to explore Taiwanese nursing students’ perspectives on online teaching during the COVID-19 pandemic and whether changes in teaching models have affected their intention to join the nursing workforce. A multi-center cross-sectional survey was conducted. Nursing students at universities and those at 5-year junior colleges were recruited to participate in the study. Data were collected through an online questionnaire survey. A total of 687 students responded to the questionnaire. The results were analyzed using percentages, mean ranks, and nonparametric methods. The results showed that 78.6% of the students agreed that online teaching was more flexible; most students stated that technical problems with computer equipment and stability of the network were large challenges that impeded online teaching. Furthermore, up to 64.8% of the students considered that online courses had affected their preparations for future nursing jobs, especially in terms of a lack of proficiency in nursing skills and inadequacy in actual interactions with patients. Online teaching is a powerful tool for nursing education, but a thoughtful strategy and more proactive approach are necessary to overcome the existing challenges for online teaching.

## 1. Introduction

In 2020, the World Health Organization declared COVID-19 a pandemic [1], triggering a global health issue. Owing to the strong transmission ability and high fatality rate of COVID-19, many countries have closed schools, which has affected nearly 50% of students worldwide [2]. Inevitably, nursing education has been affected and has shifted from the traditional teaching model to online teaching.

In the past, not all nursing education programs in Taiwan were conducted using the traditional face-to-face teaching method as blended learning was also one of the commonly used teaching methods. Blended learning combines face-to-face courses and online teaching. In nursing education, blended learning has been recognized as a supplement to traditional teaching that can effectively improve students’ knowledge, satisfaction, and learning motivation [3,4,5]. After the outbreak of COVID-19 in Taiwan in 2019, the pandemic did not affect nursing education initially because of effective pandemic control. However, in May 2021, the sudden increase in the number of COVID-19 cases resulted in the closure of schools and suspension of hospital internships nationwide. All nursing education programs were suddenly moved online, posing a challenge for nursing education. Unlike blended learning in the past when students still had access to teaching at physical sites, courses that are completely online lack actual operations and limit nursing students’ access to face-to-face practice and interaction with teachers, which are the largest disadvantages. Unlike regular education, nursing education requires more technical operations and clinical practice, so that students can be well-prepared for future clinical work. However, because of the COVID-19 pandemic, nursing education programs have been moved fully online. The lack of clinical practice may lead to stressful emotions in students [6], especially senior nursing students who are about to graduate and enter the workplace [7]. The lack of confidence and proficiency in nursing skills will become the main source of stress for new nursing graduates [8,9,10,11]. Therefore, whether fully online teaching will threaten nursing students’ ability to complete their nursing education and prepare them for clinical work is an issue of great concern.

The retention of new nursing graduates in nursing jobs has proven to be a global problem in healthcare [7,12]. Fresh nursing graduates have a very high turnover rate in Taiwan. A study conducted by Cheng et al. found that the average percentage of new Taiwanese nursing graduates with a turnover intention within the first year of employment was as high as 23% to 28% [13]. Previous studies found that nursing students’ unwillingness to join the nursing workforce was correlated with job stress, job satisfaction, lack of support, problems with hospital equipment, inadequate preparation of professional skills, and anxiety about their inability to manage patient’s conditions [8,11,12,13]. New nursing graduates are limited in their ability to manage clinical emergencies and challenging patients [8,12]. The nursing skills learned at school are important tools in shaping the professional role development and clinical skills of students. The combination of skill learning at school with clinical practice experiences can influence students’ intention to pursue a nursing career in the future [14]. Nursing students must acquire knowledge and develop their professional competencies through real-world experience, which may be a challenge that affects the effectiveness of online nursing education. Therefore, if students are not fully prepared for future clinical work through clinical practice, the lack of skills in particular will affect their intention to join the nursing workforce in the future [12]. There is no doubt that the current COVID-19 pandemic has intensified the focus on online teaching, and this transition is expected to become a permanent trend in nursing education in the future. Therefore, the purpose of this study was to explore Taiwanese nursing students’ perspectives on online teaching during the COVID-19 pandemic and whether changes in teaching models would affect their intention to join the nursing workforce in the future.

## 2. Materials and Methods

### 2.1. Study Design

A multi-center cross-sectional survey was conducted to investigate the online learning experiences of nursing students at six nursing schools in Taiwan during the COVID-19 pandemic. This study was conducted through an online questionnaire survey at four universities and two 5-year junior colleges, which were located in the northern, central, and southern regions of Taiwan, and all the schools provided face-to-face teaching to nursing students before the COVID-19 pandemic. According to the government regulations of Taiwan, all schools should switch to fully online courses from May 2021 and use online software for teaching, such as Microsoft Teams, Google Meet, Moodle, or other similar tools. All the schools were equipped with an information center, which could help students and teachers use these digital technologies and software. In this study, the data collection period was from October 2021 to January 2022. The report of this study followed the strengthening the reporting of observational studies in epidemiology (STROBE) guidelines.

### 2.2. Participants and Recruitment

The participants were nursing students in the 3rd or 4th year in general universities and nursing students in the 4th or 5th year of 5-year junior colleges in Taiwan because students at this stage started learning nursing techniques and began clinical practice. In Taiwan, nursing education is divided into two levels: nursing education in general universities and 5-year nursing programs in junior colleges. The department of nursing in a general university offers a 4-year program, and that in a 5-year junior college offers a 5-year program. Both programs include the learning of nursing techniques and clinical practice, which are started in the third and fourth grades at general universities and in the fourth and fifth grades at junior colleges. Students aged 18 years or above who had learned nursing techniques and clinical practice during the online teaching period and could read and understand Mandarin were allowed to participate in this study, but students in graduate programs were excluded because most of them had already participated in clinical work and obtained relevant licenses.

### 2.3. Sample Size

All the eligible nursing students from the participating schools were invited to participate in this study. The sample size was estimated using the online Raosoft sample size calculator [15]. The sample size was calculated based on a population of 20,000, a response rate of 50%, a confidence interval of 99%, and an error range of 5%, so the required maximum sample size was 643. A convenience sample of 687 nursing students was included in this study.

### 2.4. Measures

We developed a new questionnaire for this study. The questionnaire was drafted by the researcher following a literature search and review. The content validity was examined through expert judgment. Five teachers with a doctor’s degree who were currently engaged in nursing education were invited to conduct the expert review. After the questionnaire was revised according to expert opinions, five students in the third and fourth grades from other healthcare-related departments, apart from the nursing department, were invited to provide comments on the readability and understandability of the questionnaire. The five students belonged to the same age group as the participants, and their learning courses had also been moved online. The questionnaire was revised again according to the feedback from the students who participated in the pretest of the survey.

The questionnaire consisted of 28 structured questions and 4 open-ended questions. The structured questionnaire in the first section covered nursing students’ attitudes toward the online courses (12 items); their abilities to operate the equipment and computer provided for online courses and to solve related problems (7 items); their perspectives on achieving the learning objective through the online course (6 items); and their intentions to engage in clinical nursing work in the future (3 items). The content validity index value of each scale was 0.92–0.96. The questionnaire adopted the 5-point Likert scale, which was scored as follows: 1 point: Strongly Disagree; 2 points: Disagree; 3 points: Neither Agree nor Disagree; 4 points: Agree; and 5 points: Strongly Agree. A higher score indicated that the student has a more positive attitude toward the online courses, better computer equipment and operation skills, a stronger agreement that the learning objective can be effectively achieved through online teaching, and a stronger intention to engage in clinical nursing work in the future. The questionnaire also recorded the demographic characteristics of the participants, such as age, gender, length of schooling, year in school, region (northern, central, and southern), and the online teaching modes currently used by the school: synchronous teaching (the teacher gives a live lesson and teaching material), asynchronous (the teacher pre-records the lesson), and mixed use of synchronous and asynchronous teaching (mixed use of the live and prerecorded lessons). The second section had four open-ended questions, which allowed free answers from the students. Questions included:

“Can online courses fully replace traditional face-to-face teaching during the COVID-19 pandemic? Why?”

“What are the advantages of the online nursing courses?”

“What are the disadvantages of the online nursing courses? How to improve these disadvantages?”

“What are the effects of online courses on your learning and intention to engage in the clinical nursing work in the future?”

### 2.5. Data Collection

Data were collected through an online questionnaire created using Google Forms. The first section of the structured questionnaires could be submitted only after all the questions were answered, so the data collected have no missing values. The questionnaire began with a short paragraph describing the purpose of our study, the eligibility criteria for participants, the notification of informed consent, and an explanation to the participants that all data would be collected anonymously. A teacher from the nursing department of each participating school is the site personnel of the school, who assists in sending the standardized email to the school email accounts of students. Each email contained a link to the questionnaire. The reminder email would be sent to students every 3 days for a total of three times after the standardized email was sent. Repetition questionnaires were restricted based on IP address to ensure that only one questionnaire was completed by one person.

### 2.6. Ethical Consideration

This study was approved by the Research Ethics Committee of a China Medical University Hospital in Taiwan (CMUH110-REC1-153). The participant was not required to provide written informed consent. All students were at 18 years or above, so consent to participate from their parents/guardians was not needed. They were asked to read informed consent on the fore page of the questionnaires before filling out the questionnaire. All the study data were collected anonymously. Once students click the “Start” button, he/she was assumed to have read the information about the study and have voluntarily agreed to participate in the study.

### 2.7. Statistical Analysis

After the questionnaires were collected, SPSS 22.0 statistical software package was used to document, decode, and analyze the data. The mean, standard deviation, percentage, and average rank were used to describe the demographic characteristics of the participants and the spread of scores for each part of the questionnaire. The Kolmogorov–Smirnov test showed that the data were not normally distributed, so the Mann–Whitney U test and Kruskal–Wallis test were conducted to determine whether there was a significant difference between individuals with different background information in their attitude toward the online courses, computer equipment and operation skills, perspective on achieving the learning objective, and intention to join the nursing workforce in the future. Spearman’s rank correlation coefficient was used to analyze the correlation among the questionnaires.

The results of the four open-ended questions were analyzed using qualitative content analysis to understand their implications. Answers to the open-ended questions were read repeatedly by two researchers to determine the initial codes, and then the entire study team held a meeting to review the codes and address the difference between the codes. Next, the data were re-examined by two researchers, and the codes were reclassified into categories. Finally, the entire team held a meeting again to review the categories and confirm the final result.

## 3. Results

A total of 699 students opened the online questionnaire. The responses of 12 students were eliminated from the analysis as the questionnaire or open-ended questions was not completely answered. The final sample included 687 nursing students (response rate was 98%).

### 3.1. Analysis of Differences between Demographic Characteristics of Participants and Various Scales

As shown in Table 1, the participants had a mean age of 20.3 (±1.6) years and were predominantly identified as female (85.6%); more than 60% of the participants were nursing students from universities, with juniors being the majority (37.1%), followed by seniors (29.7%). Most of the schools were located in northern Taiwan (52.4%). The mixed synchronous and asynchronous teaching mode was used by 62% of the participating schools, followed by synchronous teaching (36.7%), and asynchronous teaching was the least used teaching mode (1.3%).

As shown in Table 1, compared with females, males had a more positive attitude toward the online courses (*Z* = 2.156, *p* = 0.031) and a more positive perspective on achieving the learning objective (*Z* = 3.595, *p* < 0.001). In addition, compared with students from 5-year junior colleges, university students had a more positive attitude toward online courses (*Z* = 3.896, *p* < 0.001), a more positive perspective on achieving the learning objectives (*Z* = 3.321, *p* = 0.001), better skills in operating the computer equipment (*Z* = 2.424, *p* = 0.015), and a stronger intention to join the nursing workforce in the future (*Z* = 3.593, *p* < 0.001). In terms of different years of school, the juniors had a more positive attitude toward the online courses (*X*^2^ = 22.408, *p* < 0.001) and a more positive perspective on achieving the learning objective (*X*^2^ = 10.604, *p* = 0.014) than the students in the fourth year of the 5-year program. In terms of the intention to join the nursing workforce in the future, the senior students had a significantly stronger intention than those in the fifth year of the 5-year program (*X*^2^ = 17.973, *p* < 0.001). In terms of the regions where schools were located, nursing students in the northern region had a less positive attitude toward the online courses (*X*^2^ = 14.232, *p* = 0.001), a less positive perspective on achieving the learning objective (*X*^2^ = 13.052, *p* = 0.001), and poorer computer equipment and operation skills than students in the central or southern regions (*X*^2^ = 7.019, *p* = 0.03). No significant differences were observed in the online teaching mode between participants with different demographic characteristics.

### 3.2. Distribution of Scores for Each Scale

To simplify the statistical analysis, we reclassified the five categories into three categories by integrating the Agree and Strongly Agree into the Agree category and integrating the Strongly Disagree and Disagree into the Disagree category. Thus, the three new categories were Agree, Neutral, and Disagree.

#### 3.2.1. Attitude toward the Online Courses

As shown in Table 2, although 43.3% of the students preferred the online courses, 273 students (39.7%) did not think that the online courses were more attractive than the traditional classroom teaching method. A total of 44.3% of the students stated that they were more inactive in the online courses, and 47.6% did not think that online courses were more effective than traditional classroom teaching. In addition, 42.5% of students believed that online courses hindered their interaction with teachers, whereas 41.6% believed it hindered their interaction with classmates, but most agreed (78.6%) that online teaching was flexible. Furthermore, 72.3% of the students did not think that online courses could completely replace traditional face-to-face teaching, and most agreed that online courses could not replace the clinical practice (79.3%) and face-to-face teaching of nursing techniques (80.8%). Up to 64.8% of the students believed that online courses had affected their preparations for future nursing jobs.

#### 3.2.2. Computer Equipment and Operation Skills

As shown in Table 3, most of the students believed that they were competent enough to operate a computer (78.9%) and troubleshoot relevant problems (61.1%). More than 80% had a computer (86.8%) or other equipment (86%) at home to participate in the online courses and 67.3% had a stable network at home and could watch the online courses smoothly, but only approximately 47.3% of the students agreed that the school had provided a stable and smooth platform for online courses; 59.8% agreed that the school had provided information on relevant teaching tools and software.

#### 3.2.3. Perspective on Achieving the Learning Objective

As shown in Table 4, 47.6% of the students agreed that the learning objective of professional knowledge could be achieved through online courses, whereas 23.6% disagreed. In terms of learning professional skills, 56.6% of the students disagreed that the learning objective of professional skills could be achieved through online courses. A total of 73.9% believed that the learning objectives of nursing techniques and practice could not be achieved through online courses. The same results were obtained regarding clinical practice. More than 60% of the students stated that they could not practice clinical nursing assessment (66.4%) and learn how to communicate and interact with patients in the online courses (60.3%). Overall, most of the students (57.8%) disagreed that online courses could help them achieve their learning objectives.

#### 3.2.4. Intention to Join the Nursing Workforce in the Future

Overall, most of the students had a strong intention to join the nursing workforce in the future (Table 5). Up to 90.2% of the students were willing to try nursing work after graduation, and more than 80% planned (85.8%) or determined (84.9%) to join the nursing workforce after graduation.

### 3.3. Correlation Analysis of Each Scale

As shown in Table 6, the students with a more positive attitude toward online courses have better computer equipment (ρ = 0.391, *p* < 0.001) and operation skills and a stronger agreement that the learning objective can be achieved through online teaching (ρ = 0.727, *p* < 0.001). The students with better computer equipment and operation skills also have a stronger agreement that the learning objective can be achieved through online teaching (ρ = 0.322, *p* < 0.001). Moreover, the students’ attitude toward online courses will not affect their intention to engage in the clinical nursing work in the future (ρ= −0.045, *p* = 0.242). However, students who have better computer equipment and operation skills (ρ = 0.250, *p* < 0.001) and disagree that online teaching can help them grasp learning objectives effectively (ρ= −0.134, *p* < 0.001) have a stronger intent to engage in clinical nursing work in the future.

### 3.4. Analysis of the Responses to the Open-Ended Questions

Question 1—Can online courses fully replace traditional face-to-face teaching during the COVID-19 pandemic? Why?” (Table 7)

When asked “Can online courses completely replace traditional face-to-face teaching?” most students answered “No” and stated various reasons because online learning was clearly different from their previous learning experience. Approximately 57.6% (393/682) stated that the online courses did not provide them an opportunity to practice their technical skills and patient assessment, both of which could not be achieved without the actual operation or face-to-face learning and discussion with teachers. In addition, 47.8% (326/682) stated that online courses could not provide the clinical experiences of interaction with patients, which could improve their communication skills with patients. A student stated,

*“I just started the clinical practice when the pandemic spread. Many in-person interactions cannot be replaced by online interactions, and patient care and assessment cannot be instructed or practiced through online courses because clinical practice is really important for nursing students. Nursing techniques can’t be mastered without clinical practice.”* (No. 326)

In addition, 71.2% (486/682) of the students stated that the online course had too many technical problems such as unstable network communications and unclear images, which indirectly affected the learning tasks and teacher–student interactions. Furthermore, 68.6% (468/682) stated that good teacher–student interactions could not be achieved with online learning. Internet disconnections may occur, which can make it impossible for students to ask questions to the teacher in real time or for the teacher to be heard clearly due to the poor network signal, which was more common in synchronous online courses. The real time teacher–student interactions were worse in asynchronous online courses. A student said,

*“There are many uncertain factors online, such as Internet crash or disconnection. Network problems often occur, so I can’t communicate with the teacher. Sometimes, Internet lag occurs, resulting in a poor effect of online learning compared with face-to-face learning. That’s even worse, if the teacher has prerecorded the video, because that means the teacher has no interaction with us at all, and we do not want to ask questions.”* (No. 85)

Question 2—What are the advantages of the online nursing courses?”
Question 3—What are the disadvantages of the online nursing courses? How to improve these disadvantages?

Most nursing students (71.5%, 487/681) agreed that online courses had an advantage in improving the learning of professional knowledge as the video of online courses could be watched repeatedly and allowed students to learn repeatedly and determine the problem they encountered in learning. Moreover, it allowed the students to prepare a flexible plan for their study time (84.8%, 578/681) and make them less stressful regarding learning (51.2%, 349/681), which are also the advantages of online courses agreed by most of the students. However, convenience and flexibility may also be one of the shortcomings of online teaching. Some reported they were often distracted (62.1%, 423/681). They could not experience the formality of face-to-face courses as the online courses could be taken anywhere. A student described his feelings,

*“I fall asleep easily in the online courses. Home is not a good place for taking a class. I won’t feel that formal sense of face-to-face courses, so I can’t focus, and I’m often distracted by surrounding factors, such as mobile phone and TV. I can’t help watching them. The teacher won’t find out anyway. So the case is that the computer is on but I’m sleeping...”* (No. 526)

In addition, most students reported poor teacher-student interactions (62.5%, 425/680) and poor teaching effectiveness due to the limitations of computers and related equipment (74.2%, 505/680). The other shortcoming of online teaching was that online courses make the learning of nursing techniques very abstract (37.5%, 255/680). Nursing education places equal emphasis on theory and technique. In particular, the learning of nursing techniques requires the actual operation. Although the recordings of online courses can be played repeatedly, students have no in-person experiences of actual operations, making the learning of nursing techniques unrealistic. A student said,

*“Learning or practice of the nursing techniques online is an empty and unrealistic operation. Nursing techniques can’t be touched or seen online, so learning them online is quite abstract. Many techniques cannot be instructed or learned using videos….”* (No. 523)

Question 4—What are the effects of online courses on your learning and intention to engage in the clinical nursing work in the future?”

In terms of the intention to join the nursing workforce in the future, up to 85.7% (589/687) of the students stated that the online courses made them unskilled in nursing and would greatly affect their future nursing work. Moreover, with the lack of face-to-face interactions with patients, the students would not know how to communicate with patients in future clinical work (59.8%, 411/687). Most students showed great concern regarding their nursing career, they were worried about being unable to cope with the clinical situation (87.6%, 602/687) and requiring a longer time to adapt to the clinical nursing work (46%, 316/687).

*“With no face-to-face interactions with patients, online courses only provide a recording of demonstrations from teachers, which is far from reality. With no in-person interactions with patients, I’m worried about being unable to communicate well with patients in clinical work. I haven’t mastered the nursing techniques, which may affect my performance in the work. I will feel anxious and stressful regarding my lack of skills when becoming a nurse. I may need to spend more time adapting to clinical work in the future.”* (No. 116).

## 4. Discussion

### 4.1. Online Courses Are Suitable to Convey Professional Knowledge

The COVID-19 pandemic has resulted in significant impacts on the learning of nursing students. Although most students preferred online courses (43.3%, Table 2) and agreed that they were more flexible (78.6%, Table 2), only 47.6% agreed that the learning objective of professional knowledge could be achieved with online courses (Table 4). This finding was similar to the results of Ebner and Gegenfurtner et al. who found that online synchronous teaching was more effective than traditional face-to-face teaching in improving students’ knowledge [16]. A meta-analysis by Kyaw et al. also indicated that online teaching was as effective as traditional teaching in improving the knowledge of medical students, perhaps because students could review the online course at their own pace, thereby improving their learning performance [17]. Amir et al. found that online courses were preferred by first-year dental students because they were mainly studying the basic dental course, concept, and theory, and their acquisition of this knowledge could be enhanced by online courses [18]. According to our qualitative analysis, students stated that online courses were flexible for them to plan their study time and could help them understand theoretical and other professional knowledge as the online courses could be replayed. Therefore, the effectiveness of online learning may vary with students’ learning objectives and demands. These online courses are spatially and temporally flexible, so students can watch the courses repeatedly anywhere and at any time, which enhances their familiarity with the professional knowledge. However, the seemingly flexible and convenient online teaching mode can easily cause distractions among students. Students reported that not all places were suitable for online learning as they were often distracted by the surrounding environment. Salmani et al. and Dost et al. reported similar findings [19,20]. Distraction is an important factor that affects the effectiveness of online learning. Nursing teachers should determine how to make online courses as attractive as face-to-face courses. Live questions and answers, opinion surveys, or group discussions can be conducted, and these methods have been proven effective to encourage students to participate in online courses [21,22]. A positive teacher–student interaction can provide students with a greater sense of participation, thereby creating a more positive learning environment.

### 4.2. Online Learning of Clinical Practice and Nursing Techniques Has Poor Effectiveness

Overall, 47.6% of the students disagreed that online courses were more effective than traditional classroom teaching, and most believed that clinical practice and courses in nursing techniques could not be replaced by online courses (Table 2). Similar results were also obtained in achieving the learning objectives. More than 50% of the students disagreed that they were able to master the professional skills and practice the clinical nursing assessment through online courses, and 73.9% considered that nursing techniques could not be practiced from online learning (Table 4). This result was similar to the results of a study on medical students. Al-Balas et al. reported that up to 78.6% of medical students believed that acquiring sufficient learning in clinical medical skills was the largest challenge for online learning [23]; a study by Bączek et al. among 804 Polish medical students also confirmed that online learning was less effective than traditional face-to-face instructions in improving medical skills [24]. In our qualitative analysis, most of the students reported that online teaching made the learning of professional skills very abstract. In the absence of real patients, students must rely on their own imagination to learn from the course recordings. Nursing is a subject with equal emphasis on theory and technique. The course on clinical nursing techniques is one of the required courses for completing nursing education, and it requires the learning of many technical operations and practice. The instruction of technical operations and practice is a difficult part of the online courses.

### 4.3. Online Courses Hinder Teacher–Student Interactions

Although many studies have a positive attitude toward online learning and argue that online teaching is as effective as traditional teaching [17,25,26]. Some believe that online teaching should augment rather than replace traditional teaching because traditional face-to-face teaching can provide many opportunities for teacher–student interactions [4]. More teacher–student interactions are critical for successful online teaching as they can shorten the psychological distance, reduce psychological stress, and make students feel supported during the online learning [27]. In our study, nearly 40% of the students believed that online courses hindered teacher–student interactions, and 44.3% stated that they were more inactive during the online courses (Table 2). According to the qualitative data analysis, most of the students reported poor teacher–student interactions in online courses, which is similar to the findings of Salmani et al., Amir et al., and Dost et al. [18,19,20]. They found that online learning made teacher–student communication more difficult because a lack of face-to-face contact with the teacher had altered effective teacher–student communication, resulting in a lack of learning motivation among students. Currently, asynchronous online teaching with prerecorded courses or synchronous online demonstration and discussion is adopted in Taiwan. The lack of face-to-face instruction from nursing teachers in the actual operation and practice has hindered the formation of an effective teaching–learning relationship. Nursing teachers need to determine how to increase effective interaction with students in online teaching, which will be a focus of online teaching.

### 4.4. Lack of Practice of Skills in Communication with Patients

The lack of practice of skills in communication with patients is also a large problem facing online teaching. In our study, up to 60.3% of the students stated that they were unable to learn how to communicate and interact with patients from online courses (Table 4). According to the qualitative analysis, the students stated that their inability to practice social skills, such as how to communicate with patients, was the main disadvantage of online courses. Our findings are similar to the findings of Bączek et al. who reported that 70% of medical students reported a lack of interaction with patients in online learning [24]. With clinical practice being replaced by online teaching, nursing students lose the learning experience that they gain from real patients in the clinical environment. Clinical practice is crucial for nursing education, so it cannot be completely replaced by online learning [28]. It is plausible that the future use of virtual patients will partially solve this problem. However, there is a gap between the virtual and real-world. Therefore, the effectiveness of online learning should be further evaluated in future studies.

### 4.5. Technical Problems of Computer Equipment and Unstable Internet Connection Are Obstacles to the Effectiveness of Online Learning

The effectiveness of online learning is often limited by problems regarding network and computer equipment. Unstable Internet connections and unclear images have massive impacts on the learning of nursing students. Apart from the content design and arrangement of online courses, students’ feedback or interaction with teachers [26], familiarity with operations of computer equipment, and network stability affect the effectiveness of online learning [19,24,29]. A study by Bączek et al. found that 54% of medical students believed that technical problems regarding computer equipment were one of the main shortcomings of online learning. Unstable network connection or unskilled operation of equipment often results in a poor effectiveness of online learning [24]. According to Choudhury and Pattnaik, learners’ perceived ease of use and usefulness of online courses determine the effectiveness of learners’ online learning. The learners’ perceived usefulness of online courses will be weakened when they need to perform the actual operation or practice their skills [29].

According to our analysis, students with better computer equipment and operation skills had a more positive attitude toward online courses and had a stronger agreement that the learning objectives could be achieved through online teaching. That indicated perfect computer equipment and a stable network were important factors that affected the effectiveness of online learning. Our study also found that compared with students from 5-year junior colleges, university students had better computer equipment and operation skills. Additionally, nursing students in the northern region had poorer computer equipment and operation skills than those in the central or southern region. That indicated students in different regions or with different length of schooling had different computer equipment and problem-solving abilities. Our findings are consistent with the findings of most studies on online teaching during the COVID-19 pandemic [19,20,24,29]. Nursing teachers and students should receive the technical training required for online learning continuously, while relevant departments of schools should provide technical support for online teaching software. The network stability and popularization and other infrastructure problems will be obstacles to online teaching and should be solved in a timely manner. Indeed, with the rapid development of online teaching, the government should provide students with more computer hardware and opportunities to learn about technology. Otherwise, students will not be able to keep up with the trend of technological development and their learning rights will be affected. Therefore, diversified learning and sufficient equipment can cultivate digital competitiveness and learn more new knowledge.

### 4.6. Impact of Online Courses on Students’ Intention to Join the Nursing Workforce

Overall, most of the nursing students had a strong intention to join the nursing workforce in the future and agreed that online courses could convey professional knowledge. Although we found that the students’ attitude toward online courses did not affect their intention to join the nursing workforce in the future, those with a stronger intention tended to disagree that online teaching could help them achieve their learning objectives effectively. Up to 64.8% of the students indicated that online courses had an influence on their preparations for future nursing competencies (Table 2). This result is similar to that obtained by Puljak et al. who found that approximately 47.4% of the students in health professions reported that they felt deprived or worried about the lack of practical education, and up to 55.1% reported they were worried that the lack of practical education would have a permanent impact on their preparations for future jobs [30].

Most of the students expressed great concern about the lack of proficiency in nursing techniques and interactions with patients, which they considered necessary to prepare them for clinical work. Our findings are consistent with the results of recent studies that evaluated students’ perceptions of online courses during the pandemic [31,32]. Previous studies also reported that the difficulty in communication with patients and their families, lack of knowledge, and lack of confidence in providing safe and independent nursing for patients are the main sources of job stress for newly graduated nurses [8,9]. Oermann and Garvin identified a lack of confidence and competence in nursing skills as the main source of stress in newly graduated nurses [10]. The lack of professional clinical skills and lack of confidence in making clinical decisions may negatively affect nursing students, which is directly related to the fact that inadequate nursing education makes it impossible for students to fully prepare themselves for the role [12]. Clinical practice and nursing techniques are the keys to the professional development of a student, but they are usually difficult and challenging and require repeated practice and actual operations. At present, with limitations in the technology of online teaching, nursing students may experience anxiety and stress. They are worried about being unable to cope with the clinical situation and require a longer time to adapt to clinical nursing work due to a lack of nursing techniques and experience. Therefore, nursing teachers should urgently identify approaches to compensate for students’ lack of nursing skills and lack of experience in face-to-face interactions with patients over the past 2 years due to the COVID-19 pandemic, especially senior students who start clinical work this year. Although most students still hope to receive the traditional face-to-face instruction of technical skills and clinical practice, it is expected that online teaching will become an irresistible development trend in nursing education. In the current environment, no alternative solution can be implemented for the teaching of nursing techniques and clinical practice. Therefore, how to overcome challenges in the learning of nursing techniques and communication with patients is an urgent problem that needs to be solved by nursing teachers.

### 4.7. Limitations

One of the strengths of this study was the inclusion of a large sample of 687 nursing students from the northern, central, and southern regions of Taiwan. Additionally, nursing students across Taiwan were surveyed online, thereby minimizing the potential response bias. However, this study has some limitations. First, in terms of the regions of participants, the study included 360 students from the northern region, a greater number than that of the other regions. With sample bias, the results may be biased too. Second, 85.6% of participants were females, so the findings may be biased due to gender imbalance. Third, some questions of this survey needed to be answered by participants after a recall, which may affect their reports due to recall bias.

## 5. Conclusions

In this study, although the students agreed that online courses were suitable to convey professional knowledge and had the advantages of flexibility and convenience, they agreed that online learning could not fully replace face-to-face patient simulation education at physical locations. As technical operations and clinical practice are critical to nursing education, they cannot be fully replaced by online learning. Most nursing students disagreed that online education could replace traditional face-to-face education in training their clinical skills. Although their intention to join the nursing workforce in the future was not affected, they expressed great concern and stress over their clinical nursing skills, especially regarding the lack of proficiency in nursing techniques and lack of experience in face-to-face interactions with patients. Therefore, the strengthening of nursing techniques and social skills is a major problem for fresh graduates who are about to begin clinical work. The COVID-19 pandemic has sped up the transformation from traditional face-to-face teaching to online teaching, which may continue to be the mainstream pattern for nursing education in the future. Virtual technology has provided a viable option for online learning of professional skills. Therefore, future studies should explore whether the use of virtual patients can solve the problems of online learning in the instruction of nursing techniques and practice of communication with patients.

## Figures and Tables

**Table 1:** Analysis of differences between demographic characteristics of participants and various scales (n = 687).

Variable	n (%)	Attitude toward the Online Courses			Computer Equipment and Operation Skills		
		MR	*ρ_a_/Z _b_/X*^2^ *_c_*	P-hoc	MR	*ρ_a_/Z _b_/X*^2^ *_c_*	P-hoc
Age, Mean (SD)	20.3 (1.6)		0.055 _a_			0.006 _a_	
Gender			2.156 _b_ *			−0.566 _b_	
1. Female	588 (85.6)	337.31			345.75		
2. Male	99 (14.4)	383.76			333.60		
Academic system			3.896 _b_ ***			2.424 _b_ *	
1. Junior colleges	228 (33.2)	302.17			318.06		
2. Universities	459 (66.8)	364.78			356.88		
Year in school			22.408 _c_ ***	1 > 3		10.604 _c_ *	No difference between groups
1. 3rd year (Universities)	255 (37.1)	385.04			374.69		
2. 4rd year (Universities)	204 (29.7)	341.01			333.35		
3. 4rd year (Junior colleges)	168 (24.5)	294.61			322.91		
4. 5rd year (Junior colleges)	60 (8.7)	318.06			308.13		
Region			14.232 _c_ ***	2 > 1		7.019 _c_ *	3 > 1
1. Northern	360 (52.4)	318.19		3 > 1	327.23		
2. Central	199 (29.0)	361.93			351.46		
3. Southern	128 (18.6)	388.72			379.57		
Online teaching modes			4.940 _c_			5.831 _c_	
1. Synchronous	252 (36.7)	337.68			324.60		
2. Asynchronous	9 (1.3)	487.00			447.39		
3. Mixed	426 (62.0)	344.72			353.29		
**Variable**	**n (%)**	**Perspective on achieving the learning objective**			**Intention to join the nursing workforce in the future**		
		**MR**	***ρ_a_/Z _b_/X*^2^ *_c_***	**P-hoc**	**MR**	***ρ_a_/Z _b_/X*^2^ *_c_***	**P-hoc**
Age, Mean (SD)	20.3 (1.6)		0.043 _a_			0.146 _a_ ***	
Gender			3.595 _b_ ***			−1.498 _b_	
1. Female	588 (85.6)	332.86			348.36		
2. Male	99 (14.4)	410.16			318.09		
Academic system			3.321 _b_ ***			3.593 _b_ ***	
1. Junior colleges	228 (33.2)	308.42			307.83		
2. Universities	459 (66.8)	361.67			361.97		
Year in school			16.145 _c_ ***	1 > 3		17.973 _c_ ***	2 > 3
1. 3rd year (Universities)	255 (37.1)	379.52			345.81		
2. 4rd year (Universities)	204 (29.7)	340.58			382.17		
3. 4rd year (Junior colleges)	168 (24.5)	304.58			301.52		
4. 5rd year (Junior colleges)	60 (8.7)	316.05			325.49		
Region			13.052 _c_ ***	3 > 1		1.140 _c_	
1. Northern	360 (52.4)	321.35			338.51		
2. Central	199 (29.0)	353.35			355.76		
3. Southern	128 (18.6)	393.16			341.16		
Online teaching modes			1.177 _c_			5.624 _c_	
1. Synchronous	252 (36.7)	341.79			365.97		
2. Asynchronous	9 (1.3)	414.61			315.11		
3. Mixed	426 (62.0)	353.82			331.61		

MR: Mean Rank; P-hoc: Post hoc test; _a_ Calculated using Spearman’s Rank Correlation Coefficient; _b_ Calculated using Mann–Whitney U Test; _c_ Calculated using Kruskal–Wallis H test; * *p* < 0.05; *** *p* < 0.001.

**Table 2:** Attitude toward the online courses (n = 687).

Item	Agree n (%)	Disagree n (%)	Neutral n (%)
Compared with traditional classroom teaching, I think online courses are more effective.	222 (32.3)	327 (47.6)	138 (20.1)
Compared with traditional classroom teaching, I think online courses are more flexible for making learning arrangements.	540 (78.6)	59 (8.6)	88 (12.8)
Compared with traditional classroom teaching, I think online courses can better attract me to participate in learning	229 (33.4)	273 (39.7)	185 (26.9)
Compared with traditional classroom teaching, I am more active in online courses.	188 (27.4)	304 (44.3)	195 (28.4)
Compared with traditional classroom teaching, I prefer online courses.	297 (43.3)	226 (32.9)	164 (23.8)
I think online courses can replace actual clinical practice courses.	88 (12.8)	545 (79.3)	54 (7.9)
I think online courses can replace face-to-face courses in nursing techniques.	84 (12.2)	555 (80.8)	48 (7.0)
I think online courses can fully replace traditional face-to-face teaching.	109 (15.9)	497 (72.3)	81 (11.8)
I do not think online courses will hinder my discussions with teachers.	245 (35.7)	292 (42.5)	150 (21.8)
I do not think online courses will hinder my discussions with my classmates.	248 (36.1)	286 (41.6)	153 (22.3)
I do not think online courses will affect my ability to prepare for future nursing jobs.	120 (17.5)	445 (64.8)	122 (17.7)
So far, I am satisfied with online courses.	211 (30.7)	228 (33.2)	248 (36.1)

**Table 3:** Computer equipment and operation skills (n = 687).

Item	Agree n (%)	Disagree n (%)	Neutral n (%)
I have sufficient skills in operating video software, so I can attend online courses on my own.	542 (78.9)	53 (7.7)	92 (13.4)
Even if there is a problem with the computer equipment, I am still competent enough to solve it.	420 (61.1)	148 (21.5)	119 (17.3)
I have a stable network at home, which allows me to attend online courses smoothly.	462 (67.3)	124 (18.0)	101 (14.7)
I have computer equipment at home, so I can use it for online courses.	596 (86.8)	27 (3.9)	64 (9.3)
Apart from the computer, I have other hardware devices (such as mobile phone and iPad) for online courses.	591 (86.0)	33 (4.8)	63 (9.2)
My school has provided a stable and smooth platform for online courses.	325 (47.3)	188 (27.4)	174 (25.3)
My school has provided the information on teaching tools and software for online courses.	411 (59.8)	98 (14.3)	178 (25.9)

**Table 4:** Perspective on achieving the learning objective (n = 687).

Item	Agree n (%)	Disagree n (%)	Neutral n (%)
Online courses allow me to achieve my learning objective of professional nursing knowledge.	327 (47.6)	162 (23.6)	198 (28.8)
Online courses allow me to achieve my learning objective of professional nursing techniques.	162 (23.6)	389 (56.6)	136 (19.8)
Online courses allow me to achieve my learning objective regarding how to communicate with patients.	136 (19.8)	414 (60.3)	137 (19.9)
Online courses allow me to achieve my learning objective of operation and practice of nursing techniques.	93 (13.5)	508 (73.9)	86 (12.5)
Online courses allow me to achieve the objectives of clinical nursing practice (such as performing patient assessments, identifying nursing problems, and executing nursing care measures).	120 (17.5)	456 (66.4)	111 (16.2)
Collectively, online courses can fully help me achieve my learning objectives.	133 (19.3)	397 (57.8)	157 (22.9)

**Table 5:** Intention to join the nursing workforce in the future (n = 687).

Item	Agree n (%)	Disagree n (%)	Neutral n (%)
I am willing to try clinical nursing work after graduation.	620 (90.2)	19 (2.8)	48 (7.0)
I planned to engage in clinical nursing work after graduation.	589 (85.8)	27 (3.9)	71 (10.3)
I have determined to engage in clinical nursing work after graduation.	583 (84.9)	27 (3.9)	77 (11.2)

**Table 6:** Correlation analysis of each scale (n = 687).

	Computer Equipment	Learning Objective	Workforce Intention
Attitude	0.391 ***	0.727 ***	−0.045
Computer equipment		0.322 ***	0.250 ***
Learning objective			−0.134 ***

*** *p* < 0.001.

**Table 7:** Analysis of the responses to the open-ended questions.

Open-Ended Question	Answere	n (%)
Question 1 Can online courses fully replace traditional face-to-face teaching during the COVID-19 pandemic?	The online courses did not provide an opportunity to practice their technical skills and patient assessment.	393/682 (57.6)
	The online courses could not provide the clinical experiences of interaction with patients, affecting their communication skills with patients.	326/682 (47.8)
	The online course had too many technical problems such as unstable network communications and unclear images.	486/682 (71.2)
	Good teacher–student interactions could not be achieved with online learning.	468/682 (68.6)
Question 2 What are the advantages of the online nursing courses?	Online courses could be watched repeatedly and learned repeatedly.	487/681 (71.5)
	The students can prepare a flexible plan for their study time.	578/681 (84.8)
	The online course makes them less stressed regarding learning.	349/681 (51.2)
Question 3 What are the disadvantages of the online nursing courses? How to improve these disadvantages?	Poor teacher–student interactions.	425/680 (62.5)
	Poor teaching effectiveness.	505/680 (74.2)
	Online courses make the learning of nursing techniques very abstract.	255/680 (37.5)
	Lack of formality of face-to-face experience, I am often distracted.	423/681 (62.1)
Question 4 What are the effects of online courses on your learning and intention to engage in the clinical nursing work in the future?	The online courses made me unskilled in nursing skills.	589/687 (85.7)
	I don’t know how to communicate with patients.	411/687 (59.8)
	I worry about my inability to cope with clinical situations.	602/687 (87.6)
	I think I need a longer time to adapt to clinical nursing work.	316/687 (46)

## Data Availability

The datasets used and analyzed during the present study are available from the corresponding author on reasonable request.
